# Supplementary material for: Effectiveness of intravenous albumin therapy to prevent spontaneous bacterial peritonitis, renal dysfunction and death in adults with cirrhosis: a protocol for a systematic review
Source: BMJ Open. 2019 Jan 29;9(1):e025664. doi: 10.1136/bmjopen-2018-025664 (PMC6352793; doi:10.1136/bmjopen-2018-025664)
Supplement: Author's manuscript [file bmjopen-2018-025664.draft_revisions.pdf]

BMJ Open is committed to open peer review. As part of this commitment we make the peer review history of every article we publish publicly available.

When an article is published we post the peer reviewers' comments and the authors' responses online. We also post the versions of the paper that were used during peer review. These are the versions that the peer review comments apply to.

The versions of the paper that follow are the versions that were submitted during the peer review process. They are not the versions of record or the final published versions. They should not be cited or distributed as the published version of this manuscript.

BMJ Open is an open access journal and the full, final, typeset and author-corrected version of record of the manuscript is available on our site with no access controls, subscription charges or pay-per-view fees (http://bmjopen.bmj.com).

If you have any questions on BMJ Open's open peer review process please email info.bmjopen@bmj.com

BMJ Open

Effectiveness of intravenous albumin therapy to prevent spontaneous bacterial peritonitis, renal dysfunction and death in adults with cirrhosis: A protocol for a systematic review

Journal:	*BMJ Open*
Manuscript ID	bmjopen-2018-025664
Article Type:	Protocol
Date Submitted by the Author:	26-Jul-2018
Complete List of Authors:	Härmälä, Suvi; University College London, Institute of Health Informatics<br>Parisinos, Constantinos; University College London, Institute of Health Informatics<br>Ryan, Jennifer; Royal Free London NHS Foundation Trust<br>O'Brien, Alastair; UCL, Division of Medicine
Keywords:	Hepatology < INTERNAL MEDICINE, Decompensated cirrhosis, INFECTIOUS DISEASES, Albumin, Spontaneous bacterial peritonitis, Renal dysfunction

SCHOLARONE™ Manuscripts

For peer review only

EFFECTIVENESS OF INTRAVENOUS ALBUMIN THERAPY TO PREVENT SPONTANEOUS BACTERIAL PERITONITIS, RENAL DYSFUNCTION AND DEATH IN ADULTS WITH CIRRHOSIS: A PROTOCOL FOR A SYSTEMATIC REVIEW

Suvi Härmälä[1], Constantinos Parisinos[1], Jennifer Ryan[2] & Alastair O'Brien[3]

[1] Institute of Health Informatics, University College London, London, United Kingdom.

[2] Royal Free London NHS Foundation Trust, London, United Kingdom

[3] Division of Medicine, University College London, London, United Kingdom.

Corresponding author: Suvi Härmälä, Institute of Health Informatics, University College London, 222 Euston Road, London NW1 2DA, suvi.harmala.14@ucl.ac.uk, +44 (0)203 549 5338

For peer review only

ABSTRACT

Introduction Use of albumin therapy is recommended for management of disease complications in cirrhosis. The effectiveness of albumin to prevent specific disease complications and death, however, is less clear.

Methods and analysis We will search Medline (Ovid), Embase (Ovid), Cochrane Hepato-Biliary Controlled Trials Register and Cochrane Central Register of Controlled Trials for published reports on randomised controlled trials and observational studies on the effectiveness of intravenous albumin therapy to prevent spontaneous bacterial peritonitis, renal dysfunction and death in cirrhotic patients. Two independent reviewers will screen the studies for eligibility, extract data and assess risk of bias and quality of evidence using Grading of Recommendations Assessment, Development and Evaluation system. Random effects meta-analyses will be performed when appropriate.

Ethics and dissemination Formal ethical approval is not required, as no primary data will be collected for this study. We aim to publish results of this study in relevant peer-reviewed medical journal or journals. Where possible, the study results will also be presented as posters or talks at relevant medical conferences and meetings.

PROSPERO registration number CRD42018100798.

For peer review only

STRENGTHS AND LIMITATIONS OF THIS STUDY:

- The results of this review may inform the formulation of strategies for prevention of cirrhosis complications, and guidelines for albumin therapy in cirrhosis.
- This study protocol follows the recommendations by the Preferred Reporting Items for Systematic Review and Meta-Analysis Protocols (PRISMA-P).
- The selection of studies, data extraction, the risk of bias and quality of evidence assessment using Grading of Recommendations Assessment, Development and Evaluation (GRADE) system will be conducted by two independent authors.
- Studies included following this review protocol may not be homogeneous in methods limiting the ability to draw reliable conclusions.
- The study methods used in included studies may not be replicable in all settings limiting the ability to fully replicate the therapy delivery in practice.

For peer review only

BACKGROUND

More than 45 million people globally have cirrhosis and other severe forms of chronic liver disease,[1]. Driven by upward trend in obesity and alcohol consumption, the prevalence of liver disease is increasing. Chronic liver disease, due to fibrosis, usually progresses silently and slowly until the functioning of the liver is severely compromised and patients develop life-threatening complications such as ascites, spontaneous bacterial peritonitis (SBP) and renal dysfunction.

Albumin, the most abundant protein in human serum, has an important role in both maintaining fluid distribution in the body and potentially regulating immune response by binding and inactivating pro-inflammatory molecules. In advanced liver disease, the synthesis of albumin in the liver is disturbed and both the quantity and functionality of albumin are substantially reduced,[2]. Treating patients with advanced liver disease with albumin infusions might improve both their ability to respond to infectious threats such as SBP and ability to restore adequate renal blood flow. Current clinical guidelines for albumin use in decompensated cirrhosis recommend the use of intravenous albumin infusions for management of ascites-related symptoms and paracentesis (removal of ascitic fluid) and for the management of SBP, renal dysfunction and variceal bleeding,[3]. Routine albumin use is not recommended for the management of non-SBP infections,[3].

Rationale for the review

To avoid the potentially devastating consequences of the complications of cirrhosis, a better understanding of the effectiveness of available prevention and treatment strategies would be useful. While the use of albumin in the management of cirrhosis complications is currently recommended and widely employed, the effectiveness of albumin to prevent specific disease complications is less clear. Previous reviews have evaluated the effects of albumin therapy both in cirrhotic patients with infections (SBP and non-SBP),[4,5] and in patients with cirrhosis-related ascites undergoing paracentesis,[6,7]. The reviews of albumin

For peer review only

use in patients with infections,[4,5], however, were written more than five years ago and new studies may have been conducted since. The most recent reviews in albumin use in paracentesis, published in 2012,[7] and 2017,[6], on the other hand, have come to contradictory conclusions on the albumin's effectiveness to prevent of death after the procedure. None of the reviews included SBP as a study outcome and non-randomised studies were excluded.

The aim of this review is to improve our understanding of the effects of albumin use in cirrhosis by reviewing the currently available evidence and quantifying the effectiveness of intravenous albumin therapy to prevent specific cirrhosis complications, SBP and renal dysfunction, and death. In contrast to previous reviews, we will also consider evidence from non-randomised studies. The results of this review may be used to inform future guidelines and clinical management of decompensated cirrhosis.

OBJECTIVES

The aim of this review is to assess the effectiveness of intravenous albumin therapy to prevent SBP, renal dysfunction and death in adults with cirrhosis.

The objectives are:

- To assess the effectiveness of intravenous albumin therapy to prevent SBP in adults with cirrhosis (without SBP or non-SBP infection)
- To assess the effectiveness of intravenous albumin therapy to prevent renal dysfunction in adults with cirrhosis, with and without SBP or non-SBP infection.
- To assess the effectiveness of intravenous albumin therapy to prevent death in adults with cirrhosis, with and without SBP or non-SBP infection.

For peer review only - http://bmjopen.bmj.com/site/about/guidelines.xhtml

METHODS

This study protocol follows the recommendations by the Preferred Reporting Items for Systematic Review and Meta-Analysis Protocols (PRISMA-P) 2015,[8].

Eligibility criteria

Types of studies

We will include randomized clinical trials (RCTs) and cohort (with comparison group/s) and case–control studies that investigate the effectiveness of intravenous albumin therapy to prevent SBP in patients with cirrhosis (without SBP or non-SBP infection), or renal dysfunction and death in patients with cirrhosis, with and without SBP. We will include studies that have been published or accepted for publication in abstract form or in full. We will exclude review articles, meta-analyses, case reports, cross-sectional studies, animal studies, editorials, surveys of medical practice, clinical guidelines and any studies that have been fully or partially retracted from publication. Patients included in multiple studies will be reported only once.

Types of participants

We will include studies that enrol 18+ year-old adult patients with cirrhosis (regardless of severity or aetiology by any classification), and without obvious signs of baseline bacterial infection when an infection (SBP or non-SBP infection) is a study outcome.

Types of interventions

We will include studies that investigate the effects of intravenously administered albumin in any setting, of any dose, administration frequency and duration of therapy.

For peer review only

Types of comparators

We will include studies comparing albumin therapy to a placebo, an alternative intervention or no intervention.

Types of outcome measures

We will include studies that report on one or more of our primary outcomes and/or our secondary outcomes.

Primary outcomes

- SBP
- Renal dysfunction (hepatorenal syndrome and other forms of renal dysfunction)
- All-cause mortality

Secondary outcomes

- Non-SBP infections
- Admission to intensive care
- Adverse events (defined as any untoward medical occurrence in a patient or clinical trial participant administered a medicinal product and which does not necessarily have a causal relationship with this product. Serious adverse events include any adverse event that at any dose results in death, is life threatening, requires hospitalisation or prolongs existing hospitalisation, results in persistent or significant disability or incapacity, is a congenital anomaly or birth defect, or seriously jeopardises the participant by requiring intervention to prevent one of the above events. All other adverse events are considered non-serious.)

Information sources

Electronic searches

For peer review only

To capture all relevant studies, we plan to search the following databases:

- The Cochrane Hepato-Biliary Controlled Trials Register
- Cochrane Central Register of Controlled Trials (CENTRAL)
- MEDLINE (Ovid)
- EMBASE (Ovid)

Each database will be searched separately for studies published until the date of the search and the search strategy first developed in MEDLINE will be adapted to each database interface as appropriate. Where only a study protocol of an eligible study is found in the search, we will further search for the published study results and if necessary, contact the investigators named in the protocol. We plan to also search relevant studies from the reference lists of the eligible studies identified through the electronic searches and from the previous clinical guidelines for management of patients with cirrhosis.

Search strategy

We will identify relevant articles by combining search terms for albumin, SBP, ascites and cirrhosis. The provisional search terms are listed in Table 1. We will not use filters to limit the search.

Table 1. MEDLINE (Ovid) provisional search terms

Search concept	Search terms
Albumin	1. Serum Albumin, Human/
	2. Albumins/ad, tu, th [Administration & Dosage, Therapeutic Use, Therapy]
	3. Albumin.mp.
	4. 1 or 2 or 3
SBP	5. ("spontaneous bacterial peritonitis" or SBP).mp.
Ascites	6. exp ASCITES/
	7. ascit*.mp.

For peer review only

	8. 5 or 6
Cirrhosis	9. exp Liver Cirrhosis/
	10. cirrho*.mp.
	11. 8 or 9

Study records

Data management

The search results will be uploaded into reference management software (Mendeley) to remove duplicate records of the same report. The unique records will then be uploaded into web-based, systematic review management software (DistillerSR). Both the initial abstract and title screening and the full-text review and extraction of data from the eligible studies will be performed using standardised, pre-created online forms. All forms will be piloted and revised as needed by the reviewers before starting the review.

Selection process

Articles identified through the search will be first screened by one reviewer (SH) by title only and then by two independent reviewers (SH&CP) by abstract and title. Where the study eligibility cannot be established based on the title, the record will be passed on to the title and abstract screening. Similarly, where the study eligibility is uncertain based on title and abstract, the report will be passed on to the full-text review. Records subject to disagreement over eligibility will always be included in the next screening stage until they reach the full-text review.

The full-text review will be independently completed by two independent review authors (SH&CP). Reasons for exclusion of ineligible studies will be recorded. Disagreements will be resolved by discussion and if required by consulting a third review author (AO or JR). Any uncertainties will be resolved by

For peer review only

correspondence with study investigators. Multiple reports of the same study will be collated into one and, where not possible, only the most relevant report based on our eligibility criteria will be included. The study selection process will be recorded and presented in flow diagram format according to the recommendations of Preferred Reporting Items for Systematic Reviews and Meta-Analyses (PRISMA),[9].

Data collection process

The data will be extracted independently and in duplicate by two review authors (SH&CP). Disagreement will be resolved by discussion and if required by consulting a third review author (AO or JR). Uncertainties will be resolved by correspondence with study investigators.

Data items

We will extract data on:

- Study participants: inclusion and exclusion criteria, method of recruitment/selection, and distribution of study population characteristics at baseline (sex, age, aetiology,severity and characteristics of liver disease, co-morbidities, abstinence, medication/treatment other than intervention).
- Interventions and comparators (administration dose and frequency, number of individuals in intervention and comparison group, follow-up time)
- Outcomes (definition, time points measured and reported, unit of measurement, number of outcomes, unadjusted and adjusted effect measures, covariates that the effect measures were adjusted for, missing data and reasons for missingness, statistical methods used, processes for randomisation e.g. allocation concealment)
- Study designs and methods (study type, country and setting, date of study, study duration, withdrawals).
- Study quality and study bias (as per the assessments specified below).

For peer review only - http://bmjopen.bmj.com/site/about/guidelines.xhtml

- Study funding and conflicts of interest

Effect measures will be collected in the format in which they are reported and transformed for presentation and analysis if appropriate.

Outcomes and prioritisation

Our primary non-fatal outcomes, SBP and renal dysfuntion, are common and serious cirrhosis complications. Cirrhotic patients may also experience other disease complications but given albumin is most commonly used clinically to improve blood volume and for infection treatment/control, we have prioritised this common infectious complication (SBP) and a common consequence of the blood volume imbalance in cirrhosis (renal dysfunction).

Our secondary outcomes include non-SBP infections, admission to intensive care and adverse events. If albumin is effective in preventing SBP, it may also be effective against other infections. Due to its multiple functions in the body, albumin may provide protection against other life-threatening disease complications such as hepatic encephalopathy. In this review, all other potential complications are represented by the outcome "admission to intensive care". Previously reported serious adverse events in albumin therapy do include cardiac disorders and respiratory disorders (pulmonary oedema, bleeding from gastric varices) and so it is important to evaluate the therapy in context of any adverse events that may have been observed in these studies.

Assessment of risk of bias in individual studies

We will use the Cochrane Collaborations tool,[10] to assess the risk of bias in the RCTs and the Newcastle-Ottawa scale,[11] to assess risk of bias in the observational studies. Age, sex, severity and aetiology of cirrhosis, co-morbidities, abstinence and treatment/medication other than the intervention/comparator will be considered the most important confounders in this assessment. Two review authors (SH&CP) will independently assess the

For peer review only - http://bmjopen.bmj.com/site/about/guidelines.xhtml

studies for each of the risk areas by entering a quote from the study to describe the procedures, their judgement together with a justification of the judgement into the data extraction forms. Disagreements will be resolved by discussion and if required by consulting a third review author (AO or JR). The risk of bias assessments will be presented in a figure that shows the level of risk (high, low, unclear) in the different risk areas within each individual study and in a figure that describes the proportion of studies within each risk level per risk area.

Assessment of bias in conducting the systematic review

We will conduct the systematic review following this pre-specified protocol and report any differences between the methods of the complete review and this protocol in the review.

Data synthesis

Criteria for quantitative data synthesis

We plan to carry out a formal meta-analysis only where more than a single study per outcome is identified and the study protocols and measures of treatment effect are considered similar enough to produce a meaningful pooled effect.

Measures of treatment effect

For dichotomous data, the treatment effect will be estimated and presented as a risk ratio with 95% confidence intervals (CI). For time-to-event data, we will present the results as a log hazard ratio with 95% CI.

Unit of analysis issues

The outcomes will be analysed at the level of study participants from each individual study.

For peer review only - http://bmjopen.bmj.com/site/about/guidelines.xhtml

Dealing with missing data

We will contact investigators to obtain numerical outcome data that have not been fully reported (for instance where a study is identified as an abstract only or outcomes are reported in figures only). Where possible, we will calculate missing standard deviations from other reported statistics such as confidence intervals or standard errors.

Assessment of heterogeneity

To assess heterogeneity between studies, we plan to present a forest plot for each of the review outcomes. We will then calculate the formal heterogeneity variance statistics τ^2, I^2 and the Q-statistic. We will regard heterogeneity as substantial if τ^2 is greater than 0, I^2 is more than 30% and the p-value for Q-statistic is less than 0.10. We plan to further explore the potential causes of substantial heterogeneity using meta-regression (specified below).

Quantitative data synthesis

Statistical analyses will be performed using Stata and RevMan. To account for the presence of heterogeneity, we will use random-effects meta-analysis to summarise the average effects of albumin therapy on the defined outcomes across studies. The results will be presented separately for patients without and with baseline SBP or non-SBP infection in forest plots with the average treatment effect and the estimates of τ^2 and I^2.

Subgroup analysis and investigation of heterogeneity

In case we identify an adequate number of studies (studies per explanatory variable ≥10), we plan to investigate the potential causes of heterogeneity between studies through random-effects meta-regression analyses. We will consider the following categories of explanatory variables: severity of cirrhosis, and aetiology of cirrhosis. Inclusion/exclusion of the explanatory variables in the

For peer review only - http://bmjopen.bmj.com/site/about/guidelines.xhtml

heterogeneity investigations will depend on the characteristics and design of the identified studies.

Sensitivity analysis

In case the identified studies differ in terms of risk of bias, we plan to investigate the impact of excluding studies with high/unclear risk of bias on effect estimates in sensitivity analyses. If the included studies report separately on patients with different cirrhosis aetiologies or different degrees of cirrhosis severity, we plan to also investigate the impact of excluding patient populations with different aetiologies or severity on the effect estimates.

Qualitative data synthesis

We will provide a narrative summary of the study results for all outcomes. Study characteristics (participants, interventions, comparators, outcomes, study design) of included studies will also be presented in tables categorised by outcome and by patients with and without SBP or non-SBP infection at baseline. The results for outcomes where meta-analysis will not be carried out due to insufficient homogeneity between studies, or for which we find no more than one study, will be presented in forest plots without the pooled effect estimate.

Meta-bias(es)

Assessment of reporting biases across studies

We plan to investigate reporting bias using funnel plots. If there are enough studies in the analysis (minimum 10), we will also carry out the Egger's test to assess whether there is a linear association between the study's result and its standard error.

We plan to assess selective outcome reporting bias by comparing what the study set to measure and analyse in the methods section of the study report (for

For peer review only

studies published after 2006, we will also investigate the details trial protocol if it can be identified through the WHO International Clinical Trials Registry Platform,[12] launched in 2007) with the results that were reported. Using the Outcome Reporting Bias in Trials (ORBIT) classification system,[13] we will evaluate whether the risk of selective outcome reporting bias is present and whether the risk is low or high.

Confidence in cumulative evidence

We will use the Grading of Recommendations Assessment, Development and Evaluation (GRADE) Working Group system,[14] to assess and report the overall quality of the body of evidence for each outcome studied. The within-study risk of bias (methodological quality), directness of evidence, heterogeneity, the precision of effect estimates and risk of publication bias will be independently assessed by two review authors (SH&CP).

COMPETING INTERESTS

The authors declare no competing interests.

FUNDING

This work is supported by the UK Biotechnology and Biological Sciences Research Council grant number BBSRC BB/M009513/1 to SH. Funding for DistillerSR licenses is supported by the Health Innovation Challenge fund (Wellcome Trust and Department of Health) award number 164699 to AO. The funders played no role in the development of the protocol, in writing of the report or in the decision to submit the protocol for publication.

AUTHOR CONTRIBUTIONS

For peer review only

The study was conceived by SH, CP, JR and AO. SH developed the eligibility criteria, search strategy, risk of bias assessment strategy and data extraction plan with guidance from CP, JF and AO. SH wrote the manuscript, to which all authors CP, JF and AO contributed.

PATIENT AND PUBLIC INVOLVEMENT

Patients or public were not involved in the development of this protocol.

ETHICS AND DISSEMINATION

Formal ethical approval is not required for this study, as no primary data will be collected. We will publish results of this study in relevant peer-reviewed medical journal or journals. Where possible, the study results will also be presented as posters or talks at relevant medical conferences and meetings.

PROTOCOL REGISTRATION

This systematic review protocol was registered in the International Prospective Register of Systematic Reviews (PROSPERO) on 3rd July 2018 (registration number CRD42018100798).

REFERENCES

1 GBD 2016 Disease and Injury Incidence and Prevalence Collaborators. Global Burden of Disease Results Tool. 2016.http://ghdx.healthdata.org/gbd-results-tool (accessed 4 Jul 2018).

2 Domenicali M, Baldassarre M, Giannone FA, *et al.* Posttranscriptional changes of serum albumin: clinical and prognostic significance in hospitalized patients with cirrhosis. *Hepatology* 2014;**60**:1851–60.

For peer review only

doi:http://dx.doi.org/10.1002/hep.27322

3 Angeli P, Bernardi M, Villanueva C, *et al.* EASL Clinical Practice Guidelines for the management of patients with decompensated cirrhosis. *J Hepatol* 2018;**69**:406–460. doi:https://doi.org/10.1016/j.jhep.2018.03.024

4 Salerno F, Navickis RJ, Wilkes MM. Albumin Infusion Improves Outcomes of Patients With Spontaneous Bacterial Peritonitis: A Meta-analysis of Randomized Trials. *Clin Gastroenterol Hepatol* 2013;**11**:123–30. doi:10.1016/j.cgh.2012.11.007

5 Kwok CS, Krupa L, Mahtani A, *et al.* Albumin reduces paracentesis-induced circulatory dysfunction and reduces death and renal impairment among patients with cirrhosis and infection: A systematic review and meta-analysis. *Biomed Res Int* 2013;**2013**:Article ID 295153. doi:http://dx.doi.org/10.1155/2013/295153

6 Kutting F, Schubert J, Franklin J, *et al.* Insufficient evidence of benefit regarding mortality due to albumin substitution in HCC-free cirrhotic patients undergoing large volume paracentesis. *J Gastroenterol Hepatol* 2017;**32**:327–38. doi:https://dx.doi.org/10.1111/jgh.13421

7 Bernardi M, Caraceni P, Navickis RJ, *et al.* Albumin infusion in patients undergoing large-volume paracentesis: a meta-analysis of randomized trials. *Hepatology* 2012;**55**:1172–81. doi:https://dx.doi.org/10.1002/hep.24786

8 Petticrew M, Shekelle P, Stewart LA, *et al.* Preferred reporting items for systematic review and meta-analysis protocols (PRISMA-P) 2015: elaboration and explanation. *BMJ* 2015;**349**:g7647. doi:10.1136/bmj.g7647

9 Moher D, Liberati A, Tetzlaff J, *et al.* Academia and Clinic Annals of Internal Medicine Preferred Reporting Items for Systematic Reviews and Meta-Analyses: The PRISMA Statement. *Annu Intern Med* 2009;**151**:264–9. doi:10.1371/journal.pmed1000097

10 The Cochrane Collaboration. Cochrane Handbook for Systematic Reviews of Interventions. Version 5.1.0 [updated March 2011]. 2011.http://handbook-5-1.cochrane.org/ (accessed 25 Jul 2018).

11 Wells G, Shea B, O'Connell D, *et al.* The Newcastle-Ottawa Scale (NOS) for

For peer review only

assessing the quality of nonrandomised studies in meta-analyses. http://www.ohri.ca/programs/clinical_epidemiology/oxford.asp (accessed 25 Jul 2018).

12 World Health Organization. International Clinical Trials Registry Platform Search Portal. http://apps.who.int/trialsearch/ (accessed 25 Jul 2018).

13 Kirkham JJ, Dwan KM, Altman DG, *et al.* The impact of outcome reporting bias in randomised controlled trials on a cohort of systematic reviews. *BMJ* 2010;**340**:c365. doi:10.1136/bmj.c365

14 Guyatt GH, Oxman AD, Vist GE, *et al.* GRADE: an emerging consensus on rating quality of evidence and strength of recommendations. *BMJ* 2008;**336**:924–6. doi:10.1136/bmj.39489.470347.AD

For peer review only

PRISMA-P (Preferred Reporting Items for Systematic review and Meta-Analysis Protocols) 2015 checklist: recommended items to address in a systematic review protocol*

Section and topic	Item No	Checklist item	Page
**ADMINISTRATIVE INFORMATION**			
Title:			
Identification	1a	Identify the report as a protocol of a systematic review	1
Update	1b	If the protocol is for an update of a previous systematic review, identify as such	n/a
Registration	2	If registered, provide the name of the registry (such as PROSPERO) and registration number	2,16
Authors:			
Contact	3a	Provide name, institutional affiliation, e-mail address of all protocol authors; provide physical mailing address of corresponding author	1
Contributions	3b	Describe contributions of protocol authors and identify the guarantor of the review	15-16
Amendments	4	If the protocol represents an amendment of a previously completed or published protocol, identify as such and list changes; otherwise, state plan for documenting important protocol amendments	12
Support:			
Sources	5a	Indicate sources of financial or other support for the review	15
Sponsor	5b	Provide name for the review funder and/or sponsor	15
Role of sponsor or funder	5c	Describe roles of funder(s), sponsor(s), and/or institution(s), if any, in developing the protocol	15
**INTRODUCTION**			
Rationale	6	Describe the rationale for the review in the context of what is already known	4-5
Objectives	7	Provide an explicit statement of the question(s) the review will address with reference to participants, interventions, comparators, and outcomes (PICO)	5
**METHODS**			
Eligibility criteria	8	Specify the study characteristics (such as PICO, study design, setting, time frame) and report characteristics (such as years considered, language, publication status) to be used as criteria for eligibility for the review	6-7
Information sources	9	Describe all intended information sources (such as electronic databases, contact with study authors, trial registers or other grey literature sources) with planned dates of coverage	8
Search strategy	10	Present draft of search strategy to be used for at least one electronic database, including planned limits, such that it could be repeated	8-9

For peer review only

Study records:			
Data management	11a	Describe the mechanism(s) that will be used to manage records and data throughout the review	9
Selection process	11b	State the process that will be used for selecting studies (such as two independent reviewers) through each phase of the review (that is, screening, eligibility and inclusion in meta-analysis)	9-10
Data collection process	11c	Describe planned method of extracting data from reports (such as piloting forms, done independently, in duplicate), any processes for obtaining and confirming data from investigators	10
Data items	12	List and define all variables for which data will be sought (such as PICO items, funding sources), any pre-planned data assumptions and simplifications	10-11
Outcomes and prioritization	13	List and define all outcomes for which data will be sought, including prioritization of main and additional outcomes, with rationale	11
Risk of bias in individual studies	14	Describe anticipated methods for assessing risk of bias of individual studies, including whether this will be done at the outcome or study level, or both; state how this information will be used in data synthesis	11-12
Data synthesis	15a	Describe criteria under which study data will be quantitatively synthesised	12
	15b	If data are appropriate for quantitative synthesis, describe planned summary measures, methods of handling data and methods of combining data from studies, including any planned exploration of consistency (such as I^2, Kendall's τ)	12-13
	15c	Describe any proposed additional analyses (such as sensitivity or subgroup analyses, meta-regression)	13-14
	15d	If quantitative synthesis is not appropriate, describe the type of summary planned	14
Meta-bias(es)	16	Specify any planned assessment of meta-bias(es) (such as publication bias across studies, selective reporting within studies)	14-15
Confidence in cumulative evidence	17	Describe how the strength of the body of evidence will be assessed (such as GRADE)	15

*** It is strongly recommended that this checklist be read in conjunction with the PRISMA-P Explanation and Elaboration (cite when available) for important clarification on the items. Amendments to a review protocol should be tracked and dated. The copyright for PRISMA-P (including checklist) is held by the PRISMA-P Group and is distributed under a Creative Commons Attribution Licence 4.0.**

From: Shamseer L, Moher D, Clarke M, Ghersi D, Liberati A, Petticrew M, Shekelle P, Stewart L, PRISMA-P Group. Preferred reporting items for systematic review and meta-analysis protocols (PRISMA-P) 2015: elaboration and explanation. BMJ. 2015 Jan 2;349(jan02 1):g7647.

For peer review only

BMJ Open

Effectiveness of intravenous albumin therapy to prevent spontaneous bacterial peritonitis, renal dysfunction and death in adults with cirrhosis: A protocol for a systematic review

Journal:	*BMJ Open*
Manuscript ID	bmjopen-2018-025664.R1
Article Type:	Protocol
Date Submitted by the Author:	17-Nov-2018
Complete List of Authors:	Härmälä, Suvi; University College London, Institute of Health Informatics<br>Parisinos, Constantinos; University College London, Institute of Health Informatics<br>Ryan, Jennifer; Royal Free London NHS Foundation Trust<br>O'Brien, Alastair; UCL, Division of Medicine
**Primary Subject Heading**:	Gastroenterology and hepatology
Secondary Subject Heading:	Evidence based practice, Infectious diseases, Gastroenterology and hepatology, Renal medicine
Keywords:	Hepatology < INTERNAL MEDICINE, Decompensated cirrhosis, INFECTIOUS DISEASES, Albumin, Spontaneous bacterial peritonitis, Renal dysfunction

SCHOLARONE™
Manuscripts

EFFECTIVENESS OF INTRAVENOUS ALBUMIN THERAPY TO PREVENT SPONTANEOUS BACTERIAL PERITONITIS, RENAL DYSFUNCTION AND DEATH IN ADULTS WITH CIRRHOSIS: A PROTOCOL FOR A SYSTEMATIC REVIEW

Suvi Härmälä[1], Constantinos Parisinos[1], Jennifer Ryan[2] & Alastair O'Brien[3]

[1] Institute of Health Informatics, University College London, London, United Kingdom.

[2] Royal Free London NHS Foundation Trust, London, United Kingdom

[3] Division of Medicine, University College London, London, United Kingdom.

Corresponding author: Suvi Härmälä, Institute of Health Informatics, University College London, 222 Euston Road, London NW1 2DA, suvi.harmala.14@ucl.ac.uk, +44 (0)203 549 5338

For peer review only

ABSTRACT

Introduction Use of albumin therapy is recommended for management of disease complications in cirrhosis. The effectiveness of albumin to prevent specific disease complications and death, however, is less clear.

Methods and analysis We will search Medline (Ovid), Embase (Ovid), Cochrane Hepato-Biliary Controlled Trials Register and Cochrane Central Register of Controlled Trials for published reports on randomised controlled trials and observational studies on the effectiveness of intravenous albumin therapy to prevent spontaneous bacterial peritonitis, renal dysfunction and death in cirrhotic patients. Two independent reviewers will screen the studies for eligibility, extract data and assess risk of bias and quality of evidence using Grading of Recommendations Assessment, Development and Evaluation system. Random effects meta-analyses will be performed when appropriate.

Ethics and dissemination As no primary data will be collected, a formal ethical approval is not required. We plan to publish the results of this study in a relevant peer-reviewed journal or journals. The study results may also be presented at relevant conferences and meetings.

PROSPERO registration number CRD42018100798.

For peer review only

STRENGTHS AND LIMITATIONS OF THIS STUDY:

- The broad range of outcomes included this review provide clinical practice and future guidelines a comprehensive picture of the effects of albumin therapy in cirrhosis.
- This study protocol has been developed according to the recommendations of the Preferred Reporting Items for Systematic Review and Meta-Analysis Protocols (PRISMA-P).
- The selection of studies, data extraction, the risk of bias and quality of evidence assessment using Grading of Recommendations Assessment, Development and Evaluation (GRADE) system will be conducted by two independent authors.
- The eligibility criteria used may not result in the selection of studies that are homogeneous in methods limiting the ability to draw reliable conclusions.
- Inclusion of studies regardless of the type of clinical setting or frequency of albumin delivery may limit the practical applicability of the summarised therapy effects to all clinical settings.

For peer review only

For peer review only - http://bmjopen.bmj.com/site/about/guidelines.xhtml

BACKGROUND

More than 45 million people globally have cirrhosis and other severe forms of chronic liver disease,[1]. Driven by upward trend in obesity and alcohol consumption, the prevalence of liver disease is increasing. Chronic liver disease, due to fibrosis-causing inflammation, usually progresses silently and slowly until the functioning of the liver is severely compromised and patients develop life-threatening complications such as ascites, spontaneous bacterial peritonitis (SBP) and renal dysfunction.

Albumin, the most abundant protein in human serum, has an important role in both maintaining fluid distribution in the body and potentially regulating immune response by binding and inactivating pro-inflammatory molecules. In advanced liver disease, the synthesis of albumin in the liver is disturbed and both the quantity and functionality of albumin are substantially reduced,[2]. Treating patients with advanced liver disease with albumin infusions might improve both their ability to respond to infectious threats such as SBP and ability to restore adequate renal blood flow. Current clinical guidelines for albumin use in decompensated cirrhosis recommend the use of intravenous albumin infusions for management of ascites-related symptoms and paracentesis (removal of ascitic fluid) and for the management of SBP, renal dysfunction and variceal bleeding,[3]. Routine albumin use is not recommended for the management of non-SBP infections,[3].

Rationale for the review

To avoid the potentially devastating consequences of the complications of cirrhosis, a better understanding of the effectiveness of available prevention and treatment strategies would be useful. While the use of albumin in the management of cirrhosis complications is currently recommended and widely employed, the effectiveness of albumin to prevent specific disease complications is less clear. Previous reviews have evaluated the effects of albumin therapy both in cirrhotic patients with infections (SBP and non-SBP),[4,5] and in patients with cirrhosis-related ascites undergoing paracentesis,[6,7]. The reviews of albumin use in

For peer review only - http://bmjopen.bmj.com/site/about/guidelines.xhtml

patients with infections,[4,5], however, were written more than five years ago and new studies may have been conducted since. The most recent reviews in albumin use in paracentesis, published in 2012,[7] and 2017,[6], on the other hand, have come to contradictory conclusions on the albumin's effectiveness to prevent of death after the procedure. None of the reviews included SBP as a study outcome and non-randomised studies were excluded.

The aim of this review is to improve our understanding of the effects of albumin use in cirrhosis by reviewing the currently available evidence and quantifying the effectiveness of intravenous albumin therapy to prevent specific cirrhosis complications, SBP and renal dysfunction, and death. In contrast to previous reviews, we will also consider evidence from non-randomised studies. The results of this review may be used to inform future guidelines and clinical management of decompensated cirrhosis.

OBJECTIVES

The aim of this review is to assess the effectiveness of intravenous albumin therapy to prevent SBP, renal dysfunction and death in adults with cirrhosis.

The objectives are:

- To assess the effectiveness of intravenous albumin therapy to prevent SBP in adults with cirrhosis and ascites (without SBP or non-SBP infection)
- To assess the effectiveness of intravenous albumin therapy to prevent renal dysfunction in adults with cirrhosis and ascites and/or infection (SBP or non-SBP infection)
- To assess the effectiveness of intravenous albumin therapy to prevent death in adults with cirrhosis and ascites and/or infection (SBP or non-SBP infection)

For peer review only

METHODS

This study protocol has been developed following the recommendations of the Preferred Reporting Items for Systematic Review and Meta-Analysis Protocols (PRISMA-P) 2015,[8].

Eligibility criteria

Types of studies

We will include randomized clinical trials (RCTs) and cohort (with comparison group/s) and case-control studies that investigate the effectiveness of intravenous albumin therapy in patients with cirrhosis and ascites and/or infection (SBP or non-SBP infection). We will include studies that have been published or accepted for publication in abstract form or in full. We will exclude review articles, meta-analyses, case reports, cross-sectional studies, animal studies, editorials, surveys of medical practice, clinical guidelines and studies that have been retracted from publication.

Types of participants

We will include studies that enrol 18+ year-old adult patients with cirrhosis (regardless of severity or aetiology by any classification) and ascites and/or infection (SBP or non-SBP infection), and 18+ year-old adult patients with cirrhosis and ascites without obvious signs of baseline bacterial infection when an infection (SBP or non-SBP infection) is a study outcome.

Types of interventions

For peer review only - http://bmjopen.bmj.com/site/about/guidelines.xhtml

We will include studies that investigate the effects of intravenously administered albumin in any setting, of any dose, administration frequency and duration of therapy.

Types of comparators

We will include studies comparing albumin therapy to a placebo, an alternative intervention or no intervention.

Types of outcome measures

We will include studies that report on one or more of our primary outcomes and/or our secondary outcomes.

Primary outcomes

- SBP
- Renal dysfunction (hepatorenal syndrome and other forms of renal dysfunction)
- All-cause mortality

Secondary outcomes

- Non-SBP infections
- Admission to intensive care
- Adverse events (serious adverse events include any adverse event that at any dose results in death, is life-threatening, requires hospitalisation or prolongs existing hospitalisation, results in persistent or significant disability or incapacity, is a congenital anomaly or birth defect, or seriously jeopardises the participant by requiring intervention to prevent one of the above events. All other adverse events will be considered non-serious.)

For peer review only

For peer review only - http://bmjopen.bmj.com/site/about/guidelines.xhtml

Information sources

Electronic searches

To capture all relevant studies, we plan to search the following databases:

- The Cochrane Hepato-Biliary Controlled Trials Register
- Cochrane Central Register of Controlled Trials (CENTRAL)
- MEDLINE (Ovid)
- EMBASE (Ovid)

Each database will be searched separately for studies published until the date of the search and the search strategy first developed in MEDLINE will be adapted to each database interface as appropriate. Where only a study protocol of an eligible study is found in the search, we will further search for the published study results and if necessary, contact the investigators named in the protocol. We plan to also search relevant studies from the reference lists of the eligible studies identified through the electronic searches and from the previous clinical guidelines for management of patients with cirrhosis.

Search strategy

We will identify relevant articles by combining search terms for albumin and the eligible base conditions of the study participants: cirrhosis and ascites and/or infection (the search terms for SBP will capture both studies that specify the participants as having "SBP", "non-SBP infections" and "infections other than SBP"). The provisional search terms are listed in Table 1. We will not use filters to limit the search.

Table 1. MEDLINE (Ovid) provisional search terms

Search concept	Search terms
Albumin	1. Serum Albumin, Human/

For peer review only - http://bmjopen.bmj.com/site/about/guidelines.xhtml

	2. Albumins/ad, tu, th [Administration & Dosage, Therapeutic Use, Therapy]
	3. Albumin.mp.
	4. 1 or 2 or 3
SBP	5. ("spontaneous bacterial peritonitis" or SBP).mp.
Ascites	6. exp ASCITES/
	7. ascit*.mp.
	8. 5 or 6 or 7
Cirrhosis	9. exp Liver Cirrhosis/
	10. cirrho*.mp.
	11. 9 or 10
Combined search	12. 4 and 8 and 11

Study records

Data management

Duplicate records of the same report will be remove using a reference management software (Mendeley). Report screening (both by title only and based on abstract and title), full-text review and extraction of data will be performed using a web-based, systematic review management software (DistillerSR) with standardised online forms. Prior to the review, the forms will be piloted and revised if necessary.

Selection process

Articles identified through the search will be first screened by one reviewer (SH) by title only and then by two independent reviewers (SH&CP) by abstract and title. Records with uncertain eligibility, or subject to disagreement over eligibility, will always be included in the next screening stage until they reach the full-text review.

For peer review only - http://bmjopen.bmj.com/site/about/guidelines.xhtml

The full-text review will be completed by two independent review authors (SH&CP). Disagreements over eligibility at this stage will be resolved by discussion and if required by consulting a third review author (AO or JR). Any uncertainties will be resolved by contacting the study investigators. Multiple, overlapping, or companion study reports representing the same study will be combined. If this is not possible, only the report that most closely fulfils our eligibility criteria will be included. The study selection process and reasons for excluding ineligible studies will be recorded and presented in a flow diagram,[9].

Data collection process

The data will be extracted independently and in duplicate by two review authors (SH&CP). Disagreements will be resolved by discussion and if required by consulting a third review author (AO or JR). Uncertainties will be resolved by contacting the study investigators.

Data items

The data will be extracted on:

- Study participants: inclusion and exclusion criteria, recruitment/selection method, and distribution of baseline characteristics (sex, age, aetiology,severity and characteristics of liver disease, co-morbidities, abstinence, medication/treatment other than intervention).
- Interventions and comparison treatments: dose and frequency, size of intervention and comparison groups, length of follow-up.
- Outcomes: definition, time points, number of events, units of measurement, unadjusted and adjusted effect estimates, covariates used for adjustment, quantity of missing data and reasons for missingness, statistical methods.
- Study design: type of study, country, setting, year/s and duration of study.
- Study quality and study bias (as per the assessments specified below).
- Funding and competing interests.

For peer review only - http://bmjopen.bmj.com/site/about/guidelines.xhtml

Data on outcome measures will be extracted as reported and, if appropriate, transformed for presentation and analysis.

Outcomes and prioritisation

Our primary non-fatal outcomes, SBP and renal dysfuntion, are common and serious cirrhosis complications. Cirrhotic patients may also experience other disease complications but given albumin is most commonly used clinically to improve blood volume and for infection treatment/control, we have prioritised this common infectious complication (SBP) and a common consequence of the blood volume imbalance in cirrhosis (renal dysfunction).

Our secondary outcomes include non-SBP infections, admission to intensive care and adverse events. If albumin is effective in preventing SBP, it may also be effective against other infections. Due to its multiple functions in the body, albumin may provide protection against other life-threatening disease complications such as hepatic encephalopathy. In this review, all other potential complications are represented by the outcome "admission to intensive care". Previously reported serious adverse events in albumin therapy do include cardiac disorders and respiratory disorders (pulmonary oedema, bleeding from gastric varices) and so it is important to evaluate the therapy in context of any adverse events that may have been observed in these studies. The occurrence of complications and adverse events will be assessed after the start of the albumin treatment (after the delivery of the first dose of albumin).

Assessment of risk of bias in individual studies

To assess the risk of bias in RCTs, we will use the Cochrane Collaborations tool,[10]. The Newcastle-Ottawa scale,[11] will be used to assess the risk of bias in observational studies. Age, sex, severity and aetiology of cirrhosis, co-morbidities, abstinence and treatment/medication other than the intervention/comparator will be considered the most important confounders in

For peer review only - http://bmjopen.bmj.com/site/about/guidelines.xhtml

this assessment. Two independent review authors (SH&CP) will make the risk of bias judgements together with a justification for each judgement (a direct quote from the study where possible) using standardised forms in a web-based, systematic review management software (DistillerSR). Disagreements will be resolved by discussion and if required by consulting a third review author (AO or JR). The assessments will be presented in figures that show the risk of bias in different risk areas at the level of individual studies and the risk of bias in different risk areas across the studies.

Assessment of bias in conducting the systematic review

We will report any differences between the methods of this pre-specified review protocol and the methods in conducting the complete review.

Data synthesis

Criteria for quantitative data synthesis

We plan to perform a formal meta-analysis where >1 study per outcome is identified and we consider the studies similar enough to produce a meaningful pooled effect.

Measures of treatment effect

For dichotomous data, the treatment effect will be estimated and presented as a risk ratio with 95% confidence intervals (CI). For time-to-event data, the effect will be estimated and the results presented as a log hazard ratio with 95% CI.

Unit of analysis issues

The outcomes will be analysed at the level of individual study participants.

Dealing with missing data

For peer review only - http://bmjopen.bmj.com/site/about/guidelines.xhtml

To obtain outcome data that are only partially reported (for instance where only the study abstract is available or an outcome is only reported in figure format) or are missing completely (outcome was set to be measured but was not reported on), we will contact the study investigators. Where possible, we will calculate missing standard deviations from other statistics such as confidence intervals or standard errors.

Assessment of heterogeneity

We plan to present a forest plot and calculate the formal heterogeneity variance statistics τ^2, I^2 and the Q-statistic for each of the review outcomes. We will regard heterogeneity as substantial if τ^2 is greater than 0, I^2 is more than 30% and the p-value for Q-statistic is less than 0.10. We plan to explore the potential reasons for substantial heterogeneity using meta-regression (specified below).

Quantitative data synthesis

Statistical analyses will be carried out using Stata and RevMan. To account for the presence of heterogeneity, random effects meta-analysis will be used to summarise the average effects of albumin therapy on the defined outcomes. The results will be presented separately for patients without and with baseline SBP or non-SBP infection in forest plots with the average treatment effect and the estimates of τ^2 and I^2.

Subgroup analysis and investigation of heterogeneity

We plan to investigate the potential reasons for heterogeneity through random-effects meta-regression analyses. Meta-regression will be performed in case we identify ≥10 studies per explanatory variable. Given the characteristics and design of the included studies allow it, we will consider severity of cirrhosis and aetiology of cirrhosis as the variables.

For peer review only - http://bmjopen.bmj.com/site/about/guidelines.xhtml

Sensitivity analysis

In case the identified studies differ greatly in terms of risk of bias, we plan to conduct sensitivity analyses to investigate the impact of excluding studies with high/unclear risk of bias on effect estimates. If the included studies report separately on patients with different cirrhosis aetiologies or different degrees of cirrhosis severity, we plan to also investigate the impact of excluding patient populations with different aetiologies or severity on the effect estimates.

Qualitative data synthesis

We will provide a narrative study result summary for all outcomes. Study characteristics (participants, interventions, comparators, outcomes, study design) of included studies will also be presented in tables categorised by outcome and by patients with and without SBP or non-SBP infection at baseline. For any outcomes where meta-analysis will not be carried out, the results will be presented in forest plots without the pooled effect estimate. All results will be discussed in the context of the previously published systematic reviews and meta-analyses on the effects of albumin use in cirrhosis.

Meta-bias(es)

Assessment of reporting biases across studies

We plan to investigate the presence of reporting bias using funnel plots. Formal test for the presence of reporting bias (Egger's test) will be performed where there are ≥10 studies in the analysis.

We plan to assess selective outcome reporting bias by comparing what the study set to measure and analyse with the results that were reported. Any trial protocol that can be identified will be used to aid this assessment. The presence of risk of selective outcome reporting bias will be evaluated using the Outcome Reporting Bias in Trials (ORBIT) classification system,[12].

For peer review only - http://bmjopen.bmj.com/site/about/guidelines.xhtml

Confidence in cumulative evidence

We will assess and report the overall quality of the body of evidence for each review outcome using the Grading of Recommendations Assessment, Development and Evaluation (GRADE) Working Group system,[13]. The study quality will be assessed by two independent review authors (SH&CP).

COMPETING INTERESTS

The authors declare no competing interests.

FUNDING

This work is supported by the UK Biotechnology and Biological Sciences Research Council grant number BBSRC BB/M009513/1 to SH. Funding for DistillerSR licenses is supported by the Health Innovation Challenge fund (Wellcome Trust and Department of Health) award number 164699 to AO. The funders played no role in the development of the protocol, in writing of the report or in the decision to submit the protocol for publication.

AUTHOR CONTRIBUTIONS

The study was conceived by SH, CP, JR and AO. SH developed the eligibility criteria, search strategy, risk of bias assessment strategy and data extraction plan with guidance from CP, JR and AO. SH wrote the manuscript, to which all authors CP, JR and AO contributed.

PATIENT AND PUBLIC INVOLVEMENT

Patients or public were not involved in the development of this protocol.

For peer review only

ETHICS AND DISSEMINATION

As no primary data will be collected, a formal ethical approval is not required. We plan to publish the results of this study in a relevant peer-reviewed journal or journals. The study results may also be presented at relevant conferences and meetings.

PROTOCOL REGISTRATION

This systematic review protocol was registered in the International Prospective Register of Systematic Reviews (PROSPERO) on 3rd July 2018 (registration number CRD42018100798).

REFERENCES

1 GBD 2016 Disease and Injury Incidence and Prevalence Collaborators. Global Burden of Disease Results Tool. 2016.http://ghdx.healthdata.org/gbd-results-tool (accessed 4 Jul 2018).

2 Domenicali M, Baldassarre M, Giannone FA, *et al.* Posttranscriptional changes of serum albumin: clinical and prognostic significance in hospitalized patients with cirrhosis. *Hepatology* 2014;**60**:1851–60. doi:http://dx.doi.org/10.1002/hep.27322

3 Angeli P, Bernardi M, Villanueva C, *et al.* EASL Clinical Practice Guidelines for the management of patients with decompensated cirrhosis. *J Hepatol* 2018;**69**:406–460. doi:https://doi.org/10.1016/j.jhep.2018.03.024

4 Salerno F, Navickis RJ, Wilkes MM. Albumin Infusion Improves Outcomes of Patients With Spontaneous Bacterial Peritonitis: A Meta-analysis of Randomized Trials. *Clin Gastroenterol Hepatol* 2013;**11**:123–30. doi:10.1016/j.cgh.2012.11.007

For peer review only - http://bmjopen.bmj.com/site/about/guidelines.xhtml

5 Kwok CS, Krupa L, Mahtani A, *et al.* Albumin reduces paracentesis-induced circulatory dysfunction and reduces death and renal impairment among patients with cirrhosis and infection: A systematic review and meta-analysis. *Biomed Res Int* 2013;**2013**:Article ID 295153. doi:http://dx.doi.org/10.1155/2013/295153

6 Kutting F, Schubert J, Franklin J, *et al.* Insufficient evidence of benefit regarding mortality due to albumin substitution in HCC-free cirrhotic patients undergoing large volume paracentesis. *J Gastroenterol Hepatol* 2017;**32**:327–38. doi:https://dx.doi.org/10.1111/jgh.13421

7 Bernardi M, Caraceni P, Navickis RJ, *et al.* Albumin infusion in patients undergoing large-volume paracentesis: a meta-analysis of randomized trials. *Hepatology* 2012;**55**:1172–81. doi:https://dx.doi.org/10.1002/hep.24786

8 Petticrew M, Shekelle P, Stewart LA, *et al.* Preferred reporting items for systematic review and meta-analysis protocols (PRISMA-P) 2015: elaboration and explanation. *BMJ* 2015;**349**:g7647. doi:10.1136/bmj.g7647

9 Moher D, Liberati A, Tetzlaff J, *et al.* Academia and Clinic Annals of Internal Medicine Preferred Reporting Items for Systematic Reviews and Meta-Analyses: The PRISMA Statement. *Annu Intern Med* 2009;**151**:264–9. doi:10.1371/journal.pmed1000097

10 The Cochrane Collaboration. Cochrane Handbook for Systematic Reviews of Interventions. Version 5.1.0 [updated March 2011]. 2011.http://handbook-5-1.cochrane.org/ (accessed 25 Jul 2018).

11 Wells G, Shea B, O'Connell D, *et al.* The Newcastle-Ottawa Scale (NOS) for assessing the quality of nonrandomised studies in meta-analyses. http://www.ohri.ca/programs/clinical_epidemiology/oxford.asp (accessed 25 Jul 2018).

12 Kirkham JJ, Dwan KM, Altman DG, *et al.* The impact of outcome reporting bias in randomised controlled trials on a cohort of systematic reviews. *BMJ* 2010;**340**:c365. doi:10.1136/bmj.c365

13 Guyatt GH, Oxman AD, Vist GE, *et al.* GRADE: an emerging consensus on rating quality of evidence and strength of recommendations. *BMJ*

For peer review only

2008;**336**:924–6. doi:10.1136/bmj.39489.470347.AD

For peer review only

PRISMA-P (Preferred Reporting Items for Systematic review and Meta-Analysis Protocols) 2015 checklist: recommended items to address in a systematic review protocol*

Section and topic	Item No	Checklist item	Page
**ADMINISTRATIVE INFORMATION**			
Title:			
Identification	1a	Identify the report as a protocol of a systematic review	1
Update	1b	If the protocol is for an update of a previous systematic review, identify as such	n/a
Registration	2	If registered, provide the name of the registry (such as PROSPERO) and registration number	2,16
Authors:			
Contact	3a	Provide name, institutional affiliation, e-mail address of all protocol authors; provide physical mailing address of corresponding author	1
Contributions	3b	Describe contributions of protocol authors and identify the guarantor of the review	15-16
Amendments	4	If the protocol represents an amendment of a previously completed or published protocol, identify as such and list changes; otherwise, state plan for documenting important protocol amendments	12
Support:			
Sources	5a	Indicate sources of financial or other support for the review	15
Sponsor	5b	Provide name for the review funder and/or sponsor	15
Role of sponsor or funder	5c	Describe roles of funder(s), sponsor(s), and/or institution(s), if any, in developing the protocol	15
**INTRODUCTION**			
Rationale	6	Describe the rationale for the review in the context of what is already known	4-5
Objectives	7	Provide an explicit statement of the question(s) the review will address with reference to participants, interventions, comparators, and outcomes (PICO)	5
**METHODS**			
Eligibility criteria	8	Specify the study characteristics (such as PICO, study design, setting, time frame) and report characteristics (such as years considered, language, publication status) to be used as criteria for eligibility for the review	6-7
Information sources	9	Describe all intended information sources (such as electronic databases, contact with study authors, trial registers or other grey literature sources) with planned dates of coverage	8
Search strategy	10	Present draft of search strategy to be used for at least one electronic database, including planned limits, such that it could be repeated	8-9

For peer review only

Study records:			
Data management	11a	Describe the mechanism(s) that will be used to manage records and data throughout the review	9
Selection process	11b	State the process that will be used for selecting studies (such as two independent reviewers) through each phase of the review (that is, screening, eligibility and inclusion in meta-analysis)	9-10
Data collection process	11c	Describe planned method of extracting data from reports (such as piloting forms, done independently, in duplicate), any processes for obtaining and confirming data from investigators	10
Data items	12	List and define all variables for which data will be sought (such as PICO items, funding sources), any pre-planned data assumptions and simplifications	10-11
Outcomes and prioritization	13	List and define all outcomes for which data will be sought, including prioritization of main and additional outcomes, with rationale	11
Risk of bias in individual studies	14	Describe anticipated methods for assessing risk of bias of individual studies, including whether this will be done at the outcome or study level, or both; state how this information will be used in data synthesis	11-12
Data synthesis	15a	Describe criteria under which study data will be quantitatively synthesised	12
	15b	If data are appropriate for quantitative synthesis, describe planned summary measures, methods of handling data and methods of combining data from studies, including any planned exploration of consistency (such as I^2, Kendall's τ)	12-13
	15c	Describe any proposed additional analyses (such as sensitivity or subgroup analyses, meta-regression)	13-14
	15d	If quantitative synthesis is not appropriate, describe the type of summary planned	14
Meta-bias(es)	16	Specify any planned assessment of meta-bias(es) (such as publication bias across studies, selective reporting within studies)	14-15
Confidence in cumulative evidence	17	Describe how the strength of the body of evidence will be assessed (such as GRADE)	15

*** It is strongly recommended that this checklist be read in conjunction with the PRISMA-P Explanation and Elaboration (cite when available) for important clarification on the items. Amendments to a review protocol should be tracked and dated.** **The copyright for PRISMA-P (including checklist) is held by the PRISMA-P Group and is distributed under a Creative Commons Attribution Licence 4.0.**

From: Shamseer L, Moher D, Clarke M, Ghersi D, Liberati A, Petticrew M, Shekelle P, Stewart L, PRISMA-P Group. Preferred reporting items for systematic review and meta-analysis protocols (PRISMA-P) 2015: elaboration and explanation. BMJ. 2015 Jan 2;349(jan02 1):g7647.

For peer review only
